# Columbite Single-Crystal CoV_2_O_6_ under High Pressure: An XRD and Raman Spectroscopy Study

**DOI:** 10.1021/acs.jpcc.5c02341

**Published:** 2025-05-26

**Authors:** Josu Sánchez-Martín, Pierre Bouvier, Gaston Garbarino, Samuel Gallego-Parra, Olivier Isnard, Plácida Rodríguez-Hernández, Alfonso Muñoz, Daniel Errandonea, Julio Pellicer-Porres

**Affiliations:** † Departamento de Física Aplicada-ICMUV, Universidad de Valencia, Dr. Moliner 50, Burjassot, Valencia 46100, Spain; ‡ Université Grenoble Alpes, CNRS, Institut Néel, 25 rue des Martyrs, Grenoble 38042, BP166X, France; § European Synchrotron Radiation Facility, Grenoble 38043, France; ∥ Departamento de Física, MALTA-Consolider Team, 16749 Universidad de La Laguna, San Cristóbal de La Laguna, Tenerife E-38200, Spain

## Abstract

The application of high pressure to orthorhombic columbite CoV_2_O_6_ resulted in two phase transitions, characterized by single-crystal X-ray diffraction (XRD) and Raman spectroscopy up to 54.9(1) GPa. The first phase transition, at 20.0(1) GPa, is isostructural, preserving the space group (*Pbcn*) but implies a sudden volume reduction (4.7%) and a symmetrization of VO_6_ octahedra, as well as a group shift of the vibrational modes toward higher wavenumbers. The named columbite-II structure remains stable up to 45.6(1) GPa. Beyond this pressure, a different monoclinic polymorph (space group *P*2_1_/*c*) was deduced from the high-quality diffraction data. The two observed phase transitions are reversible, but below 4.9(3) GPa, the sample mainly transforms into the previously known brannerite polymorph. The anisotropic compressibility of the lattice axis and the Birch–Murnaghan equation of state were obtained from the XRD analysis. Our experimental results are supported by density functional theory calculations.

## Graphical abstract

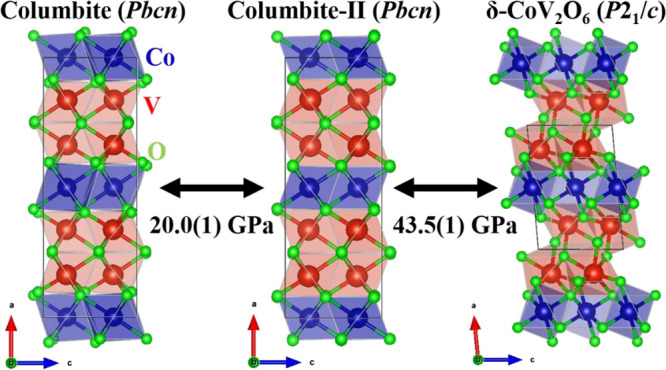

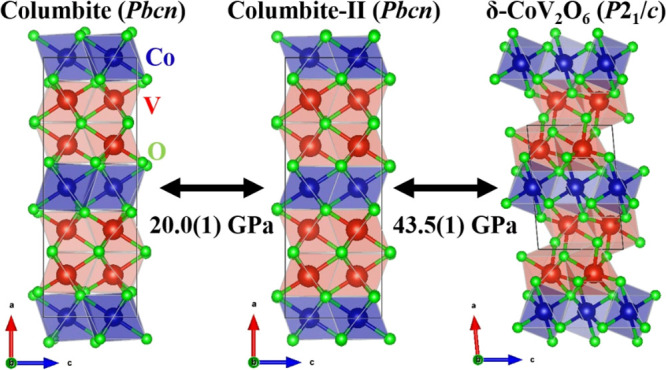

## 1 Introduction

Metastable crystal structures of compounds can be achieved either in nature or in the laboratory by means of appropriate thermodynamic conditions. The pressure–temperature conditions provide access to a relative minimum of free energy where the sample can be trapped when it is returned to ambient conditions. A compound with a given stoichiometry can, therefore, exhibit completely different physical properties depending on its crystal structure, offering future scientific opportunities.

The family of metavanadates (MV_2_O_6_, where M is a divalent metal) presents a rich variety of crystal structures depending on the metal considered, such as the most common brannerite type^1–5^ or the NiV_2_O_6_-type,^2,6,7^ among others.^1,2,6^

The monoclinic brannerite structure (space group *C*2/*m*) is the natural stable form of cobalt metavanadate. The brannerite structure has been described as having a distorted cubic close-packed oxygen network,^3,5^ although this is not strictly true. In fact, the monoclinic angle forces the distorted hexagonal oxygen planes, parallel to the (001) planes, to systematically shift in the stacking sequence. Co and V cations are located in the octahedral sites between the oxygen layers. While the Co coordination is nearly octahedral, the V coordination deviates strongly from the ideal one, to the point that its coordination with oxygens has often been described as 5 + 1. The arrangement of VO_6_ octahedra sheets is similar to that of TiO_2_ anatase, including edge-sharing two-wide zigzag chains of VO_6_ octahedrons parallel to the *b*-axis. The so-called γ-CoV_2_O_6_ polymorph has been prepared in the laboratory.^8,9^ This triclinic phase (NiV_2_O_6_-type) presents linear, edge-sharing, CoO_6_ octahedra. However, the nonequivalent V atoms in the asymmetric unit have a different coordination. While two of them maintain distorted VO_6_ coordination, the third is tetrahedrally coordinated. γ-CoV_2_O_6_ is slightly denser than brannerite.

To obtain a substantially denser polymorph, a high-pressure synthesis must be employed. In fact, it has been 50 years since a metastable subset of this family was first reported in compounds containing Ni, Mg, Co, Zn, Mn, and Cd as divalent metals. The metastable phase is obtained after applying pressures of 50–80 kbar and temperatures of 800–1200 °C and has the orthorhombic columbite-type structure (space group *Pbcn*).^10,11^ In this case, the oxygen atoms are situated in a hexagonal close packed arrangement, with cation atoms in the octahedral interstices. Along the crystallographic *c*-axis, Figure 1, there are α-PbO_2_-like columns defined by edge-sharing octahedra. The octahedra in each column are centered on either Co or V atoms. Along the *a*-axis, however, Co and V vertex-sharing octahedra alternate in a Co–V–V–Co sequence to form corrugated columns. The volume of columbite per unit formula is about 10% less than that of brannerite. Three nonequivalent positions are needed to describe the oxygens. They are color-coded in Figure 1. In a single VO_6_ octahedron, there are three O3 oxygen atoms, two O1 and one O2, while the CoO_6_ octahedra include four O2 and two O1 oxygen atoms. Along the *c*-axis, the Co octahedra are linked by oxygen atoms labeled O2, whereas O1 and O3 link V octahedra. Along the *a*-axis, the connections between Co and V octahedra are assured by the presence of O1 and O2 types of oxygen atoms. V–V octahedra are linked by O3 type oxygens.^10,11^

**Figure 1:**
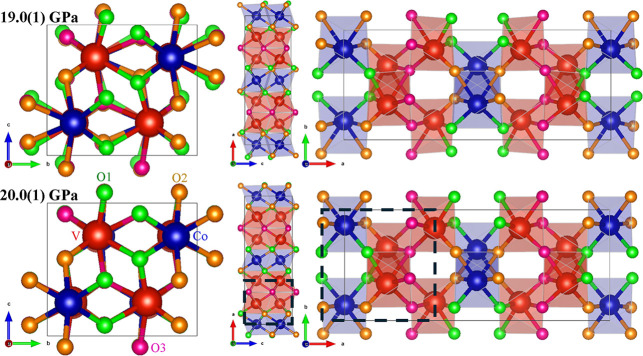
Orthorhombic (*Pbcn*) crystal structure of CoV_2_O_6_ before (19.0(1) GPa) and after (20.0(1) GPa) the isostructural phase transition. Both structures are columbite-type, but the atom positions and unit-cell parameters vary significantly. Dashed lines illustrate the relationship with the TiO_2_ (α-PbO_2_-type) phase.

With respect to high pressure (HP) characterization, only MgV_2_O_6_ has been studied in its columbite structure up to 22.5 GPa, with no phase transition detected by Raman measurements.^12^ Considering other starting structures in metavanadates, brannerite ZnV_2_O_6_ and MgV_2_O_6_ both undergo a reversible phase transition to a monoclinic structure (space group *C*2) at 16.6 ^13^ and 19 GPa,^14^ respectively. SrV_2_O_6_ and BaV_2_O_6_ transform from an orthorhombic structure (space group *C*222) to monoclinic structure (space group *C*2) at around 4 GPa, and then both suffer amorphization between 9 and 10 GPa.^15,16^ PbV_2_O_6_ remains stable in its orthorhombic structure (space group *Pnma*) up to 20 GPa.^17^ On the other hand, many more nonvanadate compounds with a columbite-type packing have been studied under HP, such as the promising photocatalysts TiO_2_,^18,19^ various niobates,^20–22^ even mixed with vanadium,^23^ or natural minerals.^24,25^

In this work, single crystals of columbite CoV_2_O_6_ have been measured up to 55 GPa by single-crystal X-ray diffraction (SCXRD) and Raman experiments, both supported by ab initio density functional theory (DFT) calculations. Two phase transitions were found at 20 and 46 GPa, the first leading to an isostructural polymorph and the second implying a monoclinic distortion (space group *P*2_1_/*c*). When the samples were released to ambient conditions, a mixed phase dominated by the brannerite polymorph was found. From the SCXRD data, the bulk modulus (B_0_) and linear compressibility were calculated for the two orthorhombic phases. The results obtained in this study make a valuable contribution to the existing body of knowledge surrounding the metavanadate family and columbite-type materials. It is further hypothesized that CoV_2_O_6_ and its rich polymorphism may exhibit technological applications that bear a notable similarity to those of titanium dioxide and niobates, which share a similar structural configuration.

## 2 Methods

### 2.1 Synthesis

Pure phase precursor powders with the same chemical formula of the product were mixed in order to prepare CoV_2_O_6_. The blend was subsequently heat treated at 700 °C for 60 min under HP of 5.5 GPa, using a Conac type press in a manner similar to that described in ref 26. This synthesis process has been followed by quenching the sample to room temperature.^11^ Single crystalline pieces of typical size 20 × 20 × 5 μm and dark color have been selected from the obtained samples.

### 2.2 Details of HP Experiments

HP experiments on single-crystal CoV_2_O_6_ were performed using a membrane-driven diamond-anvil cell (DAC) with low fluorescence, 250/300 μm beveled diamond culets. A pressure chamber of 140 μm in diameter and 35 μm in thickness was drilled in a rhenium gasket. Helium, loaded at 1.2 kbar with a gas loading system manufactured by Sanchez Technologies, was used as the pressure-transmitting medium (PTM) to ensure high hydrostatic pressure conditions up to 40 GPa,^27^ close to the highest pressure reached in this study. The pressure was measured by means of the R1-line emission of a ruby ball placed close to the sample, using IPPS-Ruby2020 equation of state.^28^ The ruby signal was measured before and after each measurement in order to control the pressure drift during long acquisitions. The recorded pressure was set at the average of these two pressure values, and the uncertainty was estimated as half of the difference between them. The homogeneity of the pressure in the DAC was followed from both the width and splitting between the R1 and R2 ruby lines.

SCXRD measurements were performed at the ID15B beamline of the ESRF^29^ using a monochromatic beam of wavelength 0.41 Å. The beam was focused down to a 1 × 1 μm^2^ full width at half-maximum. An Eiger2 X CdTe 9 M PCD device detector was used to collect the SCXRD patterns, with a sample-to-detector distance of 181 mm, which was calibrated using the SCXRD images obtained from a single-crystal vanadinite standard.^30^ The collected SCXRD images were processed with CrysAlis^Pro^ (available from Rigaku Americas Corporation), and the structural analysis was performed with OLEX2 ^31–33^ and Jana2020.^34^

The Raman measurements were performed at room temperature using a 514.54 nm laser (Cobolt Fandango) and a 750 mm spectrometer (SP2750, Acton Research) with a 1800 grooves per millimeter grating (blazed at 500 nm), equipped with a cooled charge-coupled camera (PyLoN, Princeton), and a 50 μm entrance slit size that provides a resolution of 1 cm^–1^. A set of Bragg filters (BNF-Opti grate) was used in order to reject the excitation line. The spectra were recorded in backscattering geometry with a 50× objective (Nikon) to focus the incident laser beam and collect the scattered light from inside the DAC through the diamond anvil. The wavenumber scale was calibrated using the plasma lines of a Ne–Ar lamp. The incident laser power was fixed at 8 mW (measured before the DAC) in order to avoid any laser heating of the sample. The Raman spectra covering a 15–1120 cm^–1^ spectral range were recorded using two monochromator positions, with a maximum of 240 s acquisition time averaged over two to four acquisitions. In the 25–150 cm^–1^ range, we have subtracted the contribution of N_2_/O_2_ rotations lines. Spectral parameters (position and full width at half-maximum) were obtained with MATLAB^35^ assuming a pseudo-Voigt peak profile.

### 2.3 Ab Initio Density Functional Theory Calculations

Ab initio calculations were performed within the framework of DFT^36^ using the Vienna ab initio simulation package (VASP).^37^ The projector augmented-wave method^38,39^ was employed. To ensure accurate convergence results, the plane-wave kinetic cutoff was extended up to 540 eV. The exchange–correlation energy was described via the generalized gradient approximation with the Armiento and Mattsson (AM05) prescription.^40,41^ The integrations over the Brillouin zone (BZ) were carried out with a *k*-special point sampling with a Monkhorst-Pack^42^ grid of 2 × 6 × 6. To properly treat the strongly correlated states, the DFT + *U* method of Duradev et al.^43^ was employed. This method utilizes a single parameter, *U*_eff_ = *U* – *J*, where *U* and *J* are the effective on-site Coulomb and exchange parameters, respectively. The values used for *U*_eff_ ^44^ were 3.25 eV for V and 3.32 eV for Co. Between the two structural configurations considered for the columbite-type, the antiferromagnetic one was found to be lower in energy.

The unit cell parameters and atomic positions were fully optimized to obtain, at selected volumes, a relaxed structure. For the optimization, the criteria used were that the forces on the atoms were less than 2 meV/Å and that the deviations of the stress tensors from a diagonal hydrostatic form were lower than 0.1 GPa. Our ab initio calculations provide a data set of volumes, energies, and pressures (from the stress tensor) that are fitted with a Birch–Murnaghan equation of state^45^ to obtain the theoretical equilibrium volume, the bulk modulus, and the pressure derivatives.

Lattice-dynamic calculations of the phonon modes were carried out at the zone center (Γ-point) of the BZ with the direct force-constant approach provided by the Phonopy package.^46^ These calculations provide the frequency of the normal modes, their symmetry, and their atomic displacements vectors. This allows the identification of irreducible representations and the character of the phonon modes at the Γ-point.

## 3 Results and Discussion

### 3.1 HP Single-Crystal XRD

HP SCXRD experiments show that columbite CoV_2_O_6_ remains stable at ambient temperature up to a pressure of 19.0(1) GPa. Refinement data and atomic positions at selected pressures are available in the Supporting Information (Tables S1 and S4, respectively). Beyond this pressure, the unit-cell volume decreases by 4.7%. Therefore, a first-order isostructural phase transition is observed. In addition, the positions of all atoms are slightly shifted, preserving the overall columbite arrangement but enhancing the regularity of the octahedra. The largest shift corresponds to Co. Its only atomic coordinate not determined by symmetry (*y*) changes by almost 20%. The structural information has been deposited in the Cambridge Crystallographic Data Centre (CCDC) under reference number 2387761. More refinement data and atomic positions at selected pressures are provided in the Supporting Information (Tables S2 and S5, respectively). The isostructural phase, which we will henceforth designate as “columbite-II”, is shown in Figure 1. As illustrated in the *a*-projection, the columns delineated by oxygen atoms exhibit a straighter configuration in the HP polymorph, accompanied by a reduction in polyhedral distortion. It is worth pointing out the relationship between the columbite-II phase and the TiO_2_-II (α-PbO_2_-type) structure. This phase can be considered a (slightly distorted) TiO_2_-II superstructure originating when the stacking cation planes are occupied by Co and V atoms in a Co–V–V sequence. In Figure 1, the TiO_2_-II unit cell is marked with dashed lines.

The pressure behavior of the cation environment is further analyzed in Figure 2. Panels (a) and (b) display the evolution of Co–O and V–O bonds, respectively, as well as the average bond values. In CoO_6_ octahedra, there are three different Co–O bond lengths (>2 Å). Under pressure, Co–O bonds compress homogeneously up to 20.0(1) GPa, followed by a sudden shortening of all bonds at the first phase transition. The relative average length (Δ*d*/*d*) decreases by 5.6% at the first phase transition. This jump is fairly well reproduced by ab initio calculations. VO_6_ octahedra are much more distorted. There are six different bond lengths. Three bonds are short (<1.8 Å), one of which is very short (<1.7 Å), and three long bonds (>2 Å). Long bonds compress more than short bonds up to 20.0(1) GPa, where the longest bond suddenly shortens, while the three short bonds lengthen. On average, the V–O bond does not show a marked relative jump at the first transition. The volumes of the CoO_6_ and VO_6_ octahedra in Figure 2c show that the compression of the structure is essentially supported by the larger CoO_6_ octahedron, while the VO_6_ octahedron remains rigid. At the transition at 20.0(1) GPa, the CoO_6_ octahedron shows a relative volume jump of 14.7%, while the VO_6_ volume increases slightly by 1.7%. Above 20.0(1) GPa, the compression is similar for both types of octahedra. Figure 2d shows the parameters used to assess distortion and average coordination in the different octahedra (based on the formulas in VESTA^47^). These parameters confirm that the VO_6_ octahedron is highly distorted compared with the CoO_6_ octahedron. Pressure helps to reduce the distortion of VO_6_ octahedra, while the CoO_6_ octahedra remain regular. At the first transition, there is a sudden reduction in the distortion of both octahedra. The VO_6_ octahedron is still slightly more distorted than the CoO_6_ octahedron. Cobalt coordination remains constant at 6 neighbors. Vanadium coordination is found around 3.5 and then jumps to 5 at the first transition, reaching 6 at higher pressures. This reflects the fact that the VO_6_ octahedron looks more like a square-based pyramid for the first five bonds, while the sixth is much longer. This is seen in many vanadium oxides with V^5+^ or V^4+^ as a result of vanadyl bond formation through d-p π-bonding. All of these evolutions with pressure are fairly well reproduced by ab initio calculations.

**Figure 2:**
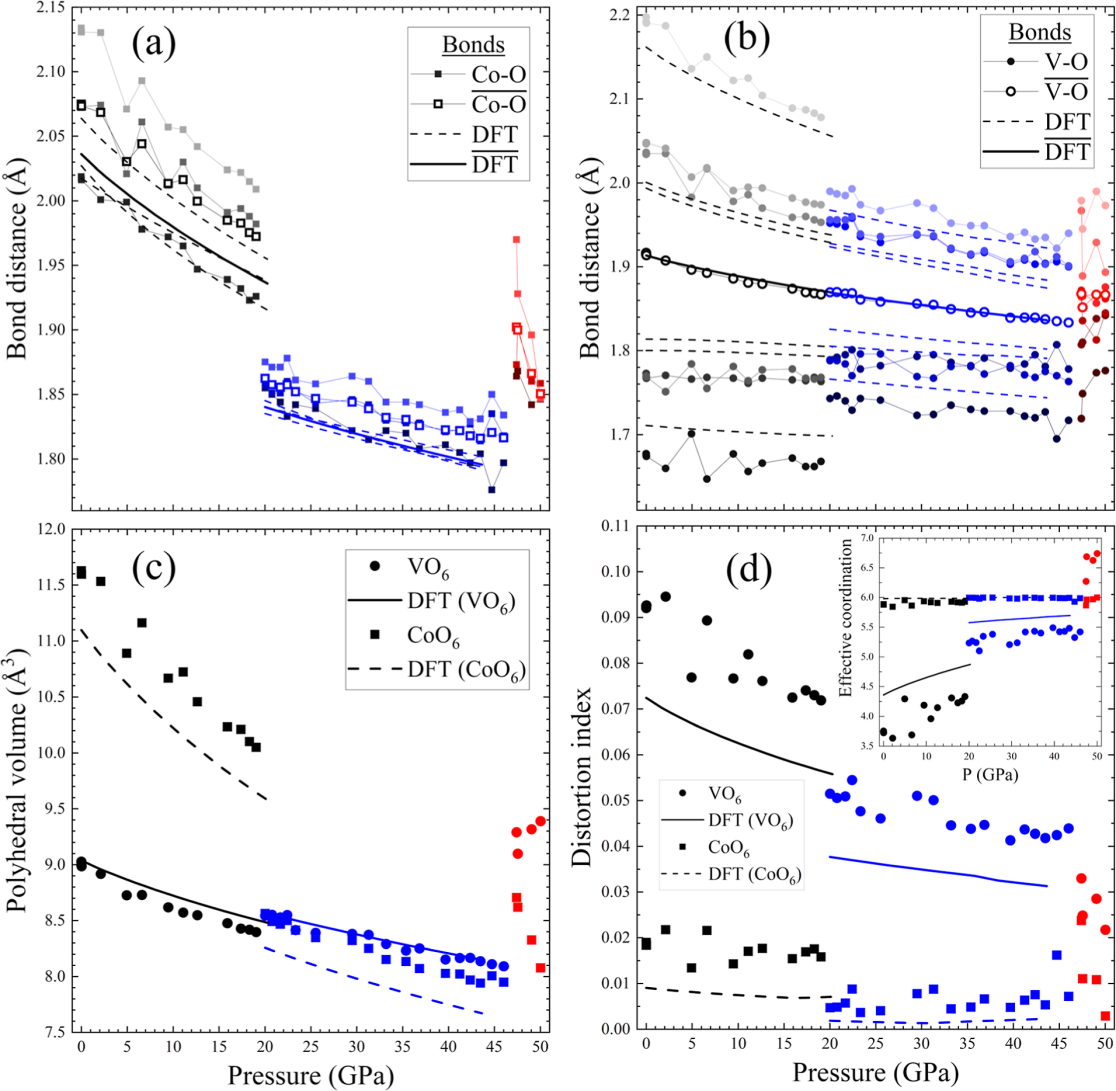
Pressure evolution of internal parameters of the CoV_2_O_6_ crystal. (a) Co–O bond distances, (b) V–O bond distances, (c) polyhedral volumes, (d) polyhedral distortion indexes, and (d, inset) effective coordination of the polyhedra.^47^ Black, blue, and red data correspond to columbite, columbite-II, and δ-CoV_2_O_6_ structures, respectively. Experimental data are shown with dots/squares (V, Co, respectively), while DFT-calculated results are represented with lines.

Taking all into account, we think that this symmetrization of the octahedra in the columbite structure could be due to the loss of its antiferromagnetic nature.^48,49^ This statement is backed by the DFT calculations reported in Figure 2, which were performed using a nonmagnetic version of the structure and remain in a fairly good agreement with the experimental results. Nevertheless, further experiments on the magnetic properties of the columbite-II structure after the phase transition are necessary to properly support this conclusion.

XRD indicates that the columbite-II structure remains stable up to 43.5(1)–46.0(1) GPa. In this pressure range, the diffraction quality drops significantly due to crystal cracking, progressively showing the increasing coexistence with a different monoclinic polymorph (space group *P*2_1_/*c*) referenced as δ-CoV_2_O_6_. The structure of this phase was solved with the greatest degree of certainty within the 47.4(1)–50.0(1) GPa pressure range. Refinement data and atomic positions extracted from the three largest pressures can be found in the Supporting Information (Tables S3 and S6, respectively). The structural information has been registered at the CCDC under deposition number 2387766. This second phase transition is associated with a 6.4% drop in unit-cell volume. The δ-CoV_2_O_6_ polymorph is represented in Figure 3. Again, the atomic arrangement is clearly related to a HP TiO_2_ polymorph, in particular the baddeleyite structured one described in ref 50. Figure 3 includes the TiO_2_ monoclinic unit cell, drawn with dashed lines. Both structures share space group. Along the *a*-axis, the TiO_2_ unit cell includes two cation planes. CoV_2_O_6_ contains three planes in a V–Co–V sequence. Consequently, the *a* lattice parameter of both compounds is related by a factor of 3/2. In both polymorphs, the oxygen atoms defining the hexagonal planes are slightly displaced from the ideal positions, with shifts occurring both along and perpendicular to the plane. As a result, the Ti and V coordination increases from 6 to 7. The associated polyhedrons define zigzag chains along the *c*-axis where the polyhedrons share edges. However, there are differences between the two structures due to the Co position, which, unlike the positions of the V and Ti atoms, is in a Wyckoff site with inversion symmetry. The resulting Co coordination is 6-fold. The connectivity between the above-mentioned zigzag chains extending along the *c*-axis is different in the monoclinic HP polymorphs of CoV_2_O_6_ and TiO_2_. The pressure evolution of the Co and V environments in the HP monoclinic phase is also included in Figure 2. It is regrettable to note that the collection of decompression data was not possible in the single-crystal XRD experiment. This was due to the collapse of the pressure chamber at the maximum pressure.

**Figure 3:**
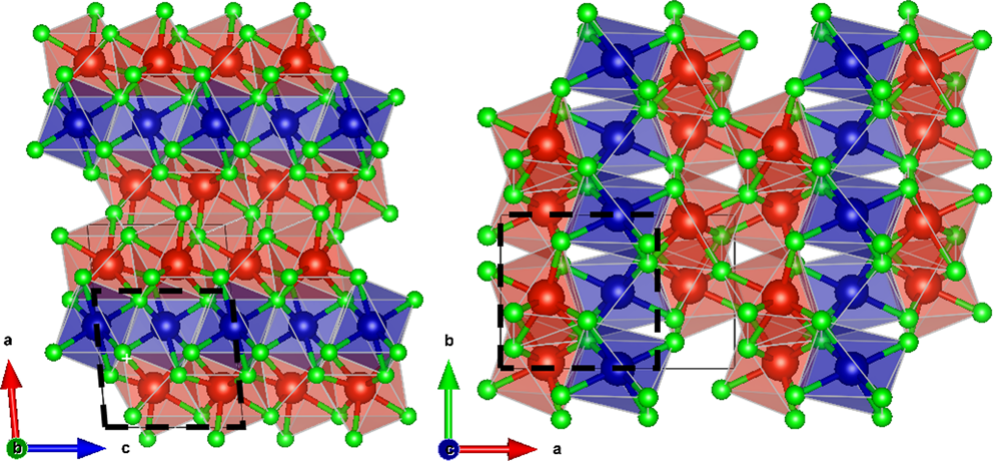
Crystal structure of monoclinic δ-CoV_2_O_6_ (space group *P*2_1_/*c*). Co, V, and O atoms are in blue, red, and green, respectively. The related monoclinic TiO_2_ unit cell is also sketched with dashed lines.

The unit-cell lattice parameters and volume of all CoV_2_O_6_ polymorphs were obtained from the structure determination of the SCXRD data up to 50.0(1) GPa. The results are presented in Figure 4 together with the DFT calculations of the columbite and columbite-II structures, showing excellent agreement. Second-order Birch–Murnaghan equations of state (EOS)^45^ were fitted using the EosFit7 software^51^ for both columbite structures (dashed lines in Figure 4). The second-order nature of the EOS was confirmed from the Eulerian strain-normalized pressure dependence of the data.^52^ The EOS fit parameters are gathered in Table 1. For experimental and calculated columbite-II data, 20.0(1) GPa was taken as the starting point for the fittings. The bulk modulus exhibited by columbite CoV_2_O_6_ (162(3) GPa) falls within the range of other metavanadates, such as the brannerite-type MgV_2_O_6_ (147(2) GPa)^14^ and ZnV_2_O_6_ (188(5) GPa).^13^

**Figure 4:**
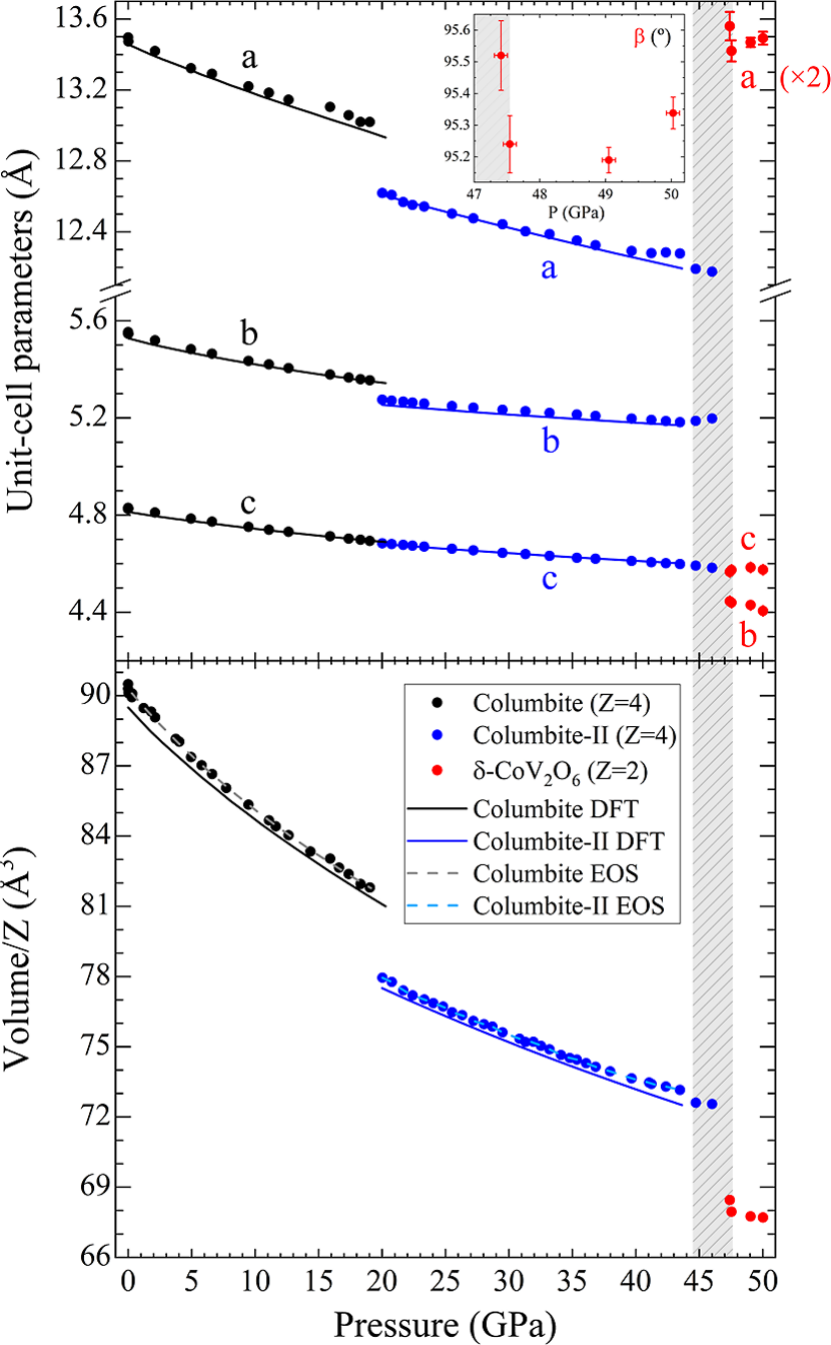
Pressure dependence of CoV_2_O_6_ unit-cell parameters (top) and volume/*Z* (bottom). Black, blue, and red dots correspond to experimental results on columbite, columbite-II, and δ-CoV_2_O_6_ polymorphs, respectively. Solid lines are DFT-calculated results, while dashed lines represent EOS fits. The shadowed region mark coexistence of columbite-II and δ-CoV_2_O_6_ phases (44.5(1) to 47.5(1) GPa). The inset shows the β parameter of the monoclinic structure. Please note that the *a* parameter of this structure is multiplied by 2.

**Table 1:** EOS Parameters (Unit-Cell Volume and Bulk Modulus) for CoV_2_O_6_ Obtained through the Fitting of Second-Order Birch–Murnaghan Model (B′ Is Fixed at 4)^a^

CoV_2_O_6_	V_0_ (Å^3^)	B_0_ (GPa)
experimental columbite	360(3)	162(3)
DFT-calculated columbite	357.8(2)	164.7(5)
experimental columbite-II (from 20 GPa)	311(2)	318(5)
DFT-calculated columbite-II (from 20 GPa)	310.0(2)	310.2(2)

a For columbite-II, V_0_ corresponds to 20 GPa.

The isothermal compressibility for all crystallographic axes of the columbite structures was obtained using the online PASCal tool.^53^ The obtained linear compressibilities are shown in Table 2 for both columbite structures as well as experimental and DFT results. All axes exhibit comparable compression parameters, with the *c*-axis displaying the least compressibility, as expected based on the edge-sharing octahedra contact along this direction (Figure 1). After the first phase transition, all axes become less affected by pressure, in particular, the *b*-axis, which more than halves its compressibility parameter and leaves the *a*-axis as the most susceptible to change under pressure (almost doubling the other two axes).

**Table 2:** Isothermal Compressibility Coefficients of the Principal Unit-Cell Axes of CoV_2_O_6_

CoV_2_O_6_ (×10^–3^ GPa^–1^)	κ_a_	κ_b_	κ_c_
experimental columbite	1.74(9)	1.80(2)	1.44(3)
DFT-calculated columbite	1.92(3)	1.63(2)	1.28(1)
experimental columbite-II (from 20 GPa)	1.22(7)	0.73(1)	0.81(1)
DFT-calculated columbite-II (from 20 GPa)	1.416(3)	0.681(2)	0.730(4)

### 3.2 HP Single-Crystal Raman Spectroscopy

As already discussed, columbite-type CoV_2_O_6_ is described by the orthorhombic space group *Pbcn*. The primitive unit cell contains 4 molecules, resulting in a total of 108 vibrational modes. At the center of the Brillouin zone, the point group symmetry (*mmm*) classifies the Raman active modes as follows^54^

$$\Gamma_{Raman}={13A}_{g}+{14B}_{1g}+{13B}_{2g}+{14B}_{3g}$$

Before starting the HP Raman experiments, we tested columbite-type CoV_2_O_6_ both in powder and in single-crystal form. The Raman spectrum obtained from the powder sample exhibited weaker Raman intensity and displayed only two additional modes compared to the spectrum obtained from the single-crystal sample. Consequently, a small single crystal was selected for loading into the DAC.

HP Raman spectra of a columbite-type CoV_2_O_6_ single crystal are shown in Figure 5. Once the sample was situated in the DAC pressure chamber, a total of 25 vibrational modes were identified in ambient conditions prior to the loading of the PTM. Three of the modes (asterisks in Figure 5) were observed again only in the 3.4(1) GPa measurement and another one in the 4.9(1) GPa downstroke pattern. Based on comparison with the spectrum of the recovered sample, see Section 3.3, we identify these modes as belonging to a residual brannerite phase.

**Figure 5:**
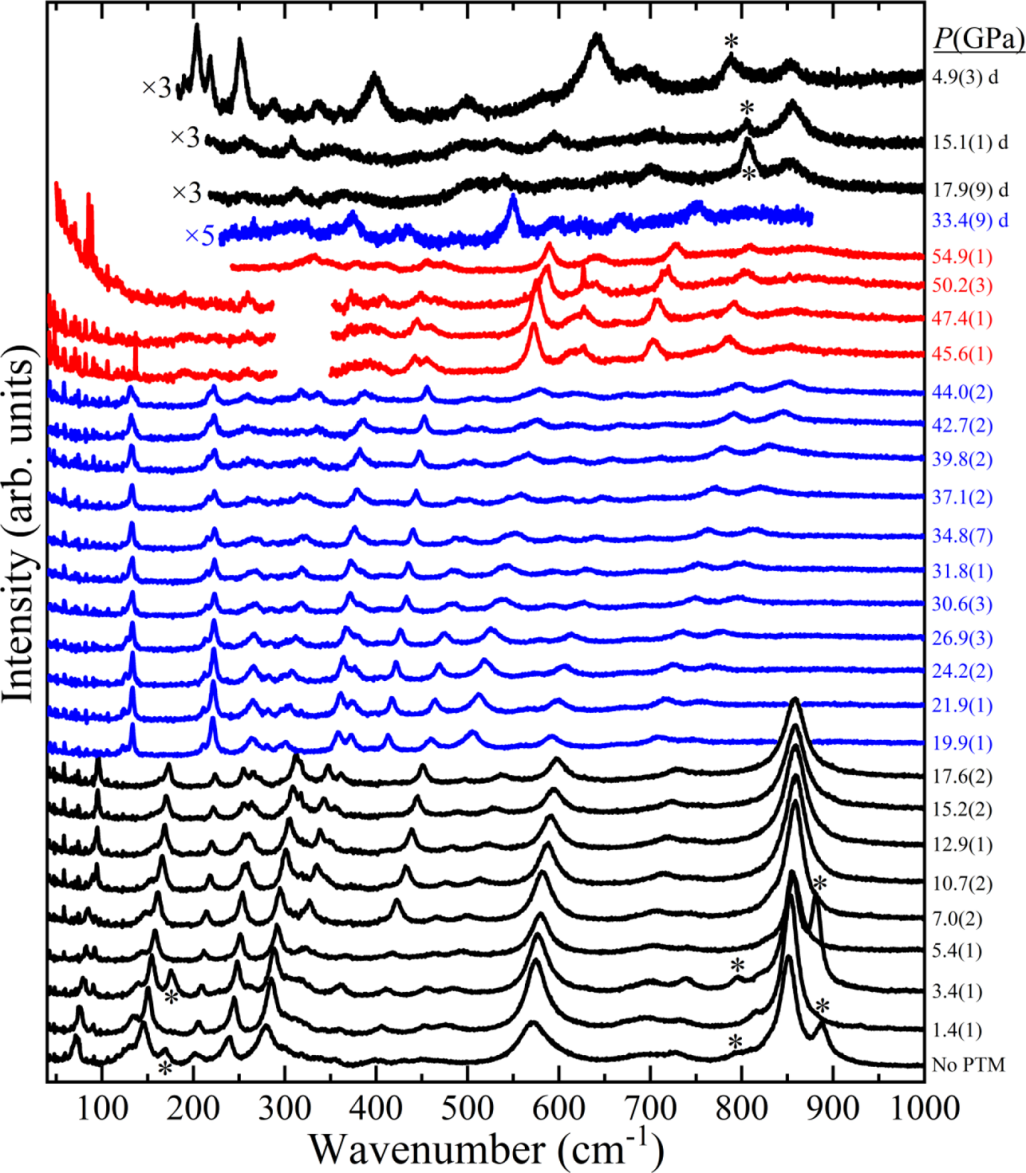
Raman spectra of single-crystal CoV_2_O_6_ at selected pressures indicated on the label to the right of the spectra. Black, blue, and red correspond to columbite, columbite-II, and monoclinic δ-CoV_2_O_6_ structures, respectively. Letter “d” indicates downstroke measurements. These spectra are zoomed to improve their visibility. Peaks labeled with asterisk belong to a residual brannerite phase.

The wavenumbers of the observed Raman modes are shown in Table 3, along with the DFT-calculated ones and their pressure coefficients. The symmetry assignment of the Raman modes is made by comparison with calculations, considering both the wavenumber and the pressure coefficient of the mode. In the absence of polarized Raman experiments, our symmetry assignment is therefore tentative. For comparison, we also present in Table 3 the mode assignment.^11^ In that work, the assignment was also not based on polarized studies but on the characterization of ZnV_2_O_6_ and the subsequent analysis of a series of compounds derived from cationic substitution. The agreement between the data presented in Table 3 is, in general, excellent.

**Table 3:** Raman Modes, Wavenumbers, and Pressure Coefficients Corresponding to the Zone-Center Raman Active Modes under Ambient Conditions for Columbite CoV_2_O_6_ ^a^

	DFT		this work		ref 11
mode	ω_0_	∂ω/∂P	ω_0_	∂ω/∂P	ω_0_
B_3g_^1^	85	2.1(1)	72(2)	2.6(2)	71 (A_g_)
A_g_^1^	100	0.8(1)			60 (A_g_)
B_3g_^2^	147	2.7(1)	129(3)	3.4(1)	130 (A_g_)
B_2g_^1^	149	1.3(1)			
B_1g_^1^	156	4.8(1)	146(2)	3.0(1)	144 (B_1g_)
B_2g_^2^	161	1.5(1)			
B_1g_^2^	162	2.7(1)			
A_g_^2^	167	1.9(1)	168(2)	3.0(7)	
B_3g_^3^	183	1.6(1)	183(5)	Powder	
B_1g_^3^	202	1.2(2)	207(4)	1.4(3)	
B_3g_^4^	208	2.0(1)	201(4)	2.1(4)	200 (B_2g,3g_)
B_2g_^3^	219	2.5(1)			
A_g_^3^	236	2.6(3)	239(3)	2.5(2)	238 (A_g_)
B_1g_^4^	236	2.7(1)	235(4)	2.7(4)	
B_3g_^5^	236	1.6(1)			
A_g_^4^	250	2.0(1)			
A_g_^5^	275	2.8(1)			
B_2g_^4^	284	2.6(1)	279(2)	2.3(1)	279 (A_g_)
B_1g_^5^	289	3.1(1)			
B_3g_^6^	292	0.8(1)			
B_2g_^5^	295	9.9(4)			
B_1g_^6^	308	0.9(1)	301(4)	1.3(4)	
B_3g_^7^	312	5.7(1)			
A_g_^6^	323	1.4(1)	317(4)	2.9(1)	320 (B_1g_)
A_g_^7^	328	2.6(1)			
B_2g_^6^	328	1.5(8)			
B_1g_^7^	330	0.9(1)	338(4)	1.0(6)	
B_2g_^7^	351	2.7(1)	354(3)	3.5(5)	356 (A_g_)
B_1g_^8^	365	2.3(1)			
A_g_^8^	367	2.4(1)			
B_3g_^8^	383	2.4(1)			
B_3g_^9^	389	4.0(1)			
B_1g_^9^	395	4.7(1)	400(3)	3.2(1)	399 (A_g_)
A_g_^9^	414	3.3(1)			
B_2g_^8^	416	1.3(1)	428(5)	1.0(5)	
B_1g_^10^	441	2.7(1)			
B_3g_^10^	447	2.8(1)	454(3)	2.6(1)	
A_g_^10^	467	5.5(1)	467(5)	5.6(1)	
B_2g_^9^	469	3.5(1)			
B_2g_^10^	477	4.5(1)			
B_3g_^11^	479	3.2(1)			
B_1g_^11^	491	5.2(1)	511(4)	Powder	
A_g_^11^	535	2.4(1)	572(2)	1.4(1)	571 (A_g_)
B_1g_^12^	565	3.6(1)			
B_3g_^12^	593	4.4(1)			
B_2g_^11^	647	2.1(1)	631(5)	2.0(5)	
A_g_^12^	666	2.5(1)			
B_1g_^13^	693	2.9(1)	697(4)	1.9(1)	692 (B_2g_)
B_3g_^13^	712	1.6(1)	727(3)	2.3(2)	723 (A_g_)
B_1g_^14^	784	3.3(1)			
B_3g_^14^	791	3.6(1)	810(4)	6.6(1)	811 (B_1g,2g,3g_)
B_2g_^12^	804	3.5(1)	830(5)	1.6(8)	
A_g_^13^	838	2.2(1)	851(2)	2.2(1)	850 (A_g_)
B_2g_^13^	842	1.4(1)			

a ω_0_ is expressed in cm^–1^, and *P*, in GPa. The DFT-calculated ω_0_ has a related uncertainty of ±5%.

When pressure was applied, 21 modes were followed up to 17.6(2) GPa. Selected Raman spectra from this range are shown in black in Figure 5, while the evolution of each mode under the pressure effects is plotted in Figure 6. All the observed modes evolve to higher wavenumbers with increasing pressure, except for the highest mode A_g_^13^ at 850 cm^–1^ (see Figure 6), which slightly softens from 10.7(2) GPa until the phase transition. This behavior is paralleled in the DFT calculations from 17.3 to 20.3 GPa. Overall, the DFT simulated modes match most of the experimental modes in both starting wavenumber at ambient conditions and pressure coefficient (see Table 3). Additionally, DFT-calculated infrared modes and their pressure coefficients can be found in Table S7.

**Figure 6:**
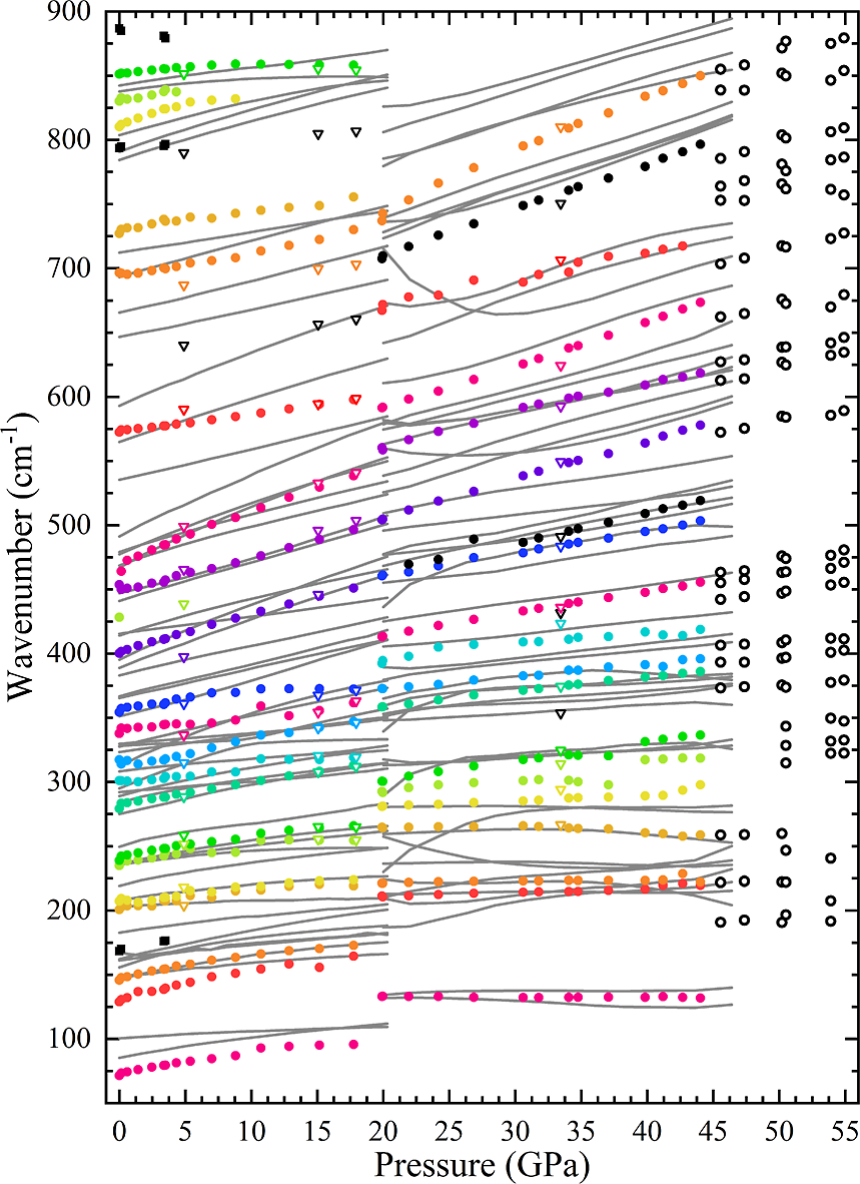
Pressure dependence of the Raman modes of columbite CoV_2_O_6_. Colored dots are experimental, while gray lines are DFT calculated. Columbite modes match in color after the first phase transition. Black squares mark brannerite CoV_2_O_6_ impurities. Additional modes after the first phase transition are in solid black, while the ones for the monoclinic δ-CoV_2_O_6_ structure are hollow black dots. Hollow triangles correspond to downstroke pressure measurements.

Continuing with the experiment, the first phase transition to the isostructural columbite-II polymorph was observed at 20.0(1) GPa (blue spectra in Figure 5). The phase transition induces the appearance of two additional modes (black dots in Figure 6) and an overall shift of the spectrum to higher wavenumbers. The shifted modes are shown in Figure 6 using the same color scheme as the columbite phase. All modes of this isostructural phase go to higher wavenumbers with increasing pressure and can be followed up to 44.0(2) GPa.

It is noticeable that the modes above 750 cm^–1^ vanish in the phase transition. We could follow the B_3g_^14^ and B_2g_^12^ modes [810(4) and 830(5) cm^–1^ under ambient conditions, respectively] only up to 9 GPa. Above this pressure, only the A_g_^13^ mode [851(2) cm^–1^ at ambient conditions] is detected. Even this very intense mode disappears in the phase transition. The transition from columbite to the columbite-II phase does not involve changes in either the symmetry or the Wyckoff position of the atoms, so the disappearance of the modes cannot be attributed to modifications of Raman selection rules induced by the phase transition. Even the symmetrized version of the columbite-II structure derived from the TiO_2_-II polymorph (see Section 3.1) shares the same space group, so arguments based on similarity to a more symmetric phase cannot be considered either. On the other hand, a HP Raman experiment on anatase TiO_2_ has been performed.^55,56^ The transition to TiO_2_-II is indeed associated with a large reduction in intensity of the more energetic modes, although they remain easily measurable.

Figure 7 shows the phonon vibration pattern corresponding to the A_g_^13^ mode in the columbite [0 GPa, panel a] and columbite-II phases [around 22 GPa, panel b]. The Co atoms remain almost static (shifts along b and c are forbidden by symmetry in this mode). The oxygen atoms with the largest amplitude vibrate quite closely in the direction of the V–O bonds, which approximately matches Co–O octahedra edges. As a result, V–O bonds stretch. Co–O bonds bend, also contributing to the restoring forces. The most significant difference between the phonon patterns of columbite and columbite-II is the motion of the V atoms, which in columbite-II has a smaller amplitude and rotates to become closer to perpendicular to the (100) stacking planes. According to calculations, the wavenumber of the A_g_^13^ mode (and also those of the closer modes) drops abruptly at the phase transition (Figure 6). We interpret the drop as a consequence of the reduction of the restoring forces induced by the changes in the V motion just described.

**Figure 7:**
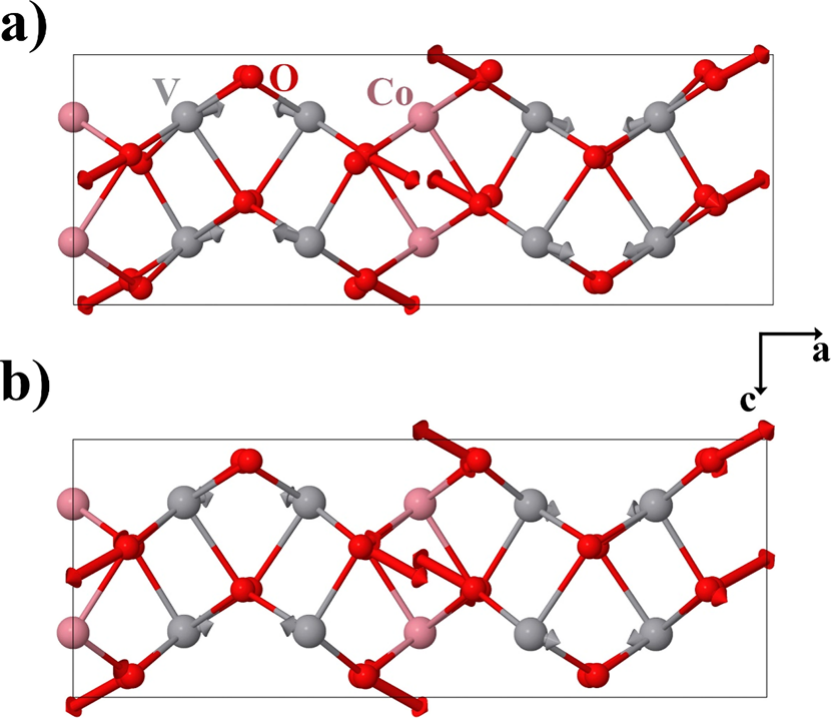
Phonon vibration pattern of the A_g_^13^ mode, following ab initio calculations. Arrow lengths are proportional to amplitude vibration. (a) Columbite phase at 0 GPa. (b) Columbite-II phase at 22 GPa.

When further pressure is applied at 45.6(1) GPa, the sample undergoes a second phase transition to monoclinic δ-CoV_2_O_6_, Figure 5. The spectrum of this phase is similar to the baddeleyite-structured TiO_2_ reported in ref 50. Since there are only two molecules in the primitive unit cell, the number of vibrational modes is halved compared to the previous phase. Of the 54 vibrational modes, 24 are Raman active (12A_g_ + 12B_g_). We were able to identify 22 modes in the measured spectra (empty black circles in Figure 6). Data dispersion is significantly increased with respect to the lower pressure polymorphs due to spectra degradation caused by the loss of crystalline quality of the sample after the phase transition.

Subsequently, measurements were conducted during the downstroke. The phonon wavenumbers derived from these spectra are represented in Figure 6 as void triangles. At a pressure of 33.4(9) GPa, the spectrum is consistent with the columbite-II structure. The polycrystalline nature of the sample in the downstroke enabled the characterization of some modes that were not observed in the upstroke (Figure 6). At a pressure of 17.9(9) GPa in the downstroke, the measured spectrum corresponds to the columbite phase. Additional modes were observed, specifically the B_2g_^11^ and A_g_^12^ modes at 640 and 687 cm^–1^, respectively. Following the HP experiment and the DAC opening, one further Raman measurement was conducted. A detailed discussion of this measurement can be found in Section 3.3.

### 3.3 Sample Recovered to Ambient Conditions

Prior to concluding the Raman experiment, the last run of measurements were obtained opening the DAC and thereby releasing all the remaining pressure. Figure 8 illustrates the Raman signal of the sample in two distinct sections (A and B). The Raman patterns exhibit a notable disparity due to the nonhomogeneous nature of the sample subsequent to the HP experiment. The fitted modes in area A (Figure 8) are consistent with the brannerite polymorph, as the Raman profile is analogous to those reported for brannerite-type ZnV_2_O_6_ ^13^ and MgV_2_O_6_.^14^ The most intense modes in Figure 8 also correspond to the extra peaks (asterisks) observed in Figure 5, indicating the presence of some brannerite CoV_2_O_6_ impurities at the beginning of the experiment. Area B demonstrates that this phase is not the sole remaining constituent, as it exhibits different vibrational modes in comparison to area A.

**Figure 8:**
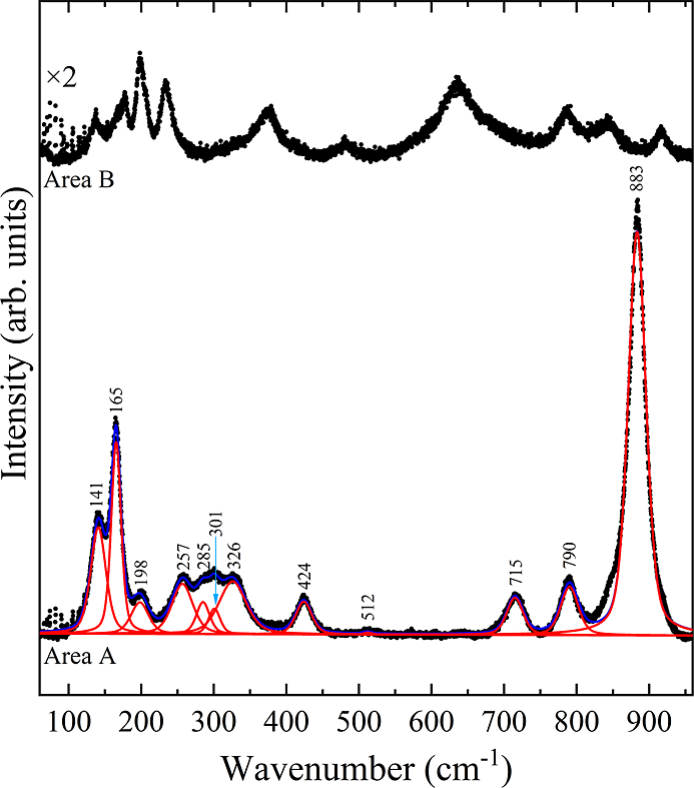
Raman spectrum of the CoV_2_O_6_ polycrystal after the HP experiment in two different sections under ambient conditions. Area A is fitted and labeled with expected modes of brannerite CoV_2_O_6_.

Additional XRD measurements were conducted on the recovered sample to identify the polymorphs present in the mixture. An attempt was made to develop an analysis similar to that presented in Section 3.1; however, the conditions of the sample precluded single-crystal treatment of the data. Consequently, all 128 diffraction images were merged into a single one and treated with the DIOPTAS software,^57^ as is customary with powder samples. Unfortunately, the data obtained from this polycrystalline sample lack the quality necessary to perform a proper powder refinement. Figure S1 of the Supporting Information shows the integrated signal of the XRD measurement matching the diffraction contributions of the potential remaining compounds in the sample. In this diffractogram, contributions from columbite CoV_2_O_6_ and Re from the gaskets are found as expected. There are also reflections of the predominant downstroke phase (brannerite) and others related to γ-CoV_2_O_6_. These two phases are to be expected as they can be synthetized at ambient pressure.^5,8^

In summary, the Raman spectra obtained following the release of all pressures indicate that brannerite-type CoV_2_O_6_ is the predominant remaining phase. The phase transition from columbite CoV_2_O_6_ occurs at pressures below 4.9(3) GPa during decompression. Although the XRD signal and its results are of a lower quality than those of the rest of this work, they might indicate the presence of residual columbite CoV_2_O_6_ and γ-CoV_2_O_6_ in the sample in addition to the predominant brannerite polymorph.

## 4 Conclusions

HP XRD and Raman spectroscopy experiments have been carried out on single crystals of columbite-type CoV_2_O_6_ up to 50.0(1) and 54.9(1) GPa, respectively. Both experiments show an isostructural phase transition to the columbite-II polymorph at 20 GPa. The XRD data reflect the symmetrization of the CoO_6_ and VO_6_ octahedra forming the structure. The Raman collective shift is due to the fact that the space group is preserved, but the volume of the structure is suddenly decreased by 4.7%, so all phonons are shifted accordingly, together with the disappearance of the modes related to the V–O bonds. At 45.6(1) GPa, the vibrational data change completely, indicating a second phase transition. The diffraction data show that this structure is a different monoclinic polymorph (space group *P*2_1_/*c*) with an increased coordination of the VO_7_ polyhedra. On decompression, both phase transitions show reversibility in the same ranges. However, below 4.9(3) GPa, the sample undergoes a further structural change, resulting in a phase admixture with CoV_2_O_6_ brannerite as the predominant polymorph. Anisotropic compressibility and EOS parameters (including bulk moduli) are obtained for both columbite polymorphs. All results for these structures are also supported by DFT calculations, which show excellent overall agreement.

## Supplementary Material



## Data Availability

The data that support the findings of this study are available from the corresponding author upon reasonable request.

## Notes

HP single-crystal XDR refinement data of columbite CoV_2_O_6_, columbite-II CoV_2_O_6_, and δ-CoV_2_O_6_; HP atomic positions determined for columbite CoV_2_O_6_, columbite-II CoV_2_O_6_, and δ-CoV_2_O_6_; DFT-calculated infrared modes at ambient conditions for columbite CoV_2_O_6_; and XRD measurement of the recovered sample after the Raman experiment on CoV_2_O_6_ (PDF)
