# Supplementary material for: Columbite Single-Crystal CoV2O6 under High Pressure: An XRD and Raman Spectroscopy Study
Source: J Phys Chem C Nanomater Interfaces. 2025 May 26;129(22):10364–74. doi: 10.1021/acs.jpcc.5c02341 (PMC12147450; doi:10.1021/acs.jpcc.5c02341)
Supplement: Supplementary file 1 [file jp5c02341_si_001.pdf]

Supporting Information

Columbite Single-Crystal CoV_2O_6 under High Pressure: An XRD and Raman Spectroscopy Study

Josu Sánchez-Martín,[†], Pierre Bouvier[‡], Gaston Garbarino[§], Samuel Gallego-Parra[§], Olivier Isnard[‡], Plácida Rodríguez-Hernández[⊥], Alfonso Muñoz[⊥], Daniel Errandonea[†] and Julio Pellicer-Porres[†]*

[†]Departamento de Física Aplicada-ICMUV, Universidad de Valencia, Dr. Moliner 50, Burjassot, 46100 Valencia, Spain. E-mail: josu.sanchez@uv.es

[‡]Université Grenoble Alpes, CNRS, Institut Néel, 25 rue des Martyrs, 38042, BP166X, Grenoble, France

[§]European Synchrotron Radiation Facility, 38043 Grenoble, France

[⊥] Departamento de Física, MALTA-Consolider Team, Universidad de La Laguna, San Cristóbal de La Laguna, E-38200 Tenerife, Spain

*josu.sanchez@uv.es

Table S1. Single crystal XDR refinement data of columbite CoV_2O_6 at selected pressures.

**Pressure (GPa)**	**0.0(1)**	**5.0(1)**	**9.5(1)**	**15.9(1)**	**19.0(1)**
Formula	CoV_2O_6	CoV_2O_6	CoV_2O_6	CoV_2O_6	CoV_2O_6
Dcalc./g·cm^{-3}	4.729	4.881	4.997	5.136	5.213
μ/mm^{-1}	2.046	2.113	2.159	2.222	2.256
Formula Weight	256.81	256.81	256.81	256.81	256.81
Colour	black	black	black	black	black
Shape	plate	plate	plate	plate	plate
Size/μm^3	20×20×5	20×20×5	20×20×5	20×20×5	20×20×5
T/K	293(2)	293(2)	293(2)	293(2)	293(2)
Crystal System	orthorhombic	orthorhombic	orthorhombic	orthorhombic	orthorhombic
Space Group	*Pbcn*	*Pbcn*	*Pbcn*	*Pbcn*	*Pbcn*
a/Å	13.474(7)	13.338(10)	13.216(10)	13.097(8)	13.011(9)
b/Å	5.5461(2)	5.4790(3)	5.4355(3)	5.3803(2)	5.3566(2)
c/Å	4.8268(2)	4.7825(3)	4.7524(3)	4.7137(2)	4.6947(2)
α/°	90	90	90	90	90
β/°	90	90	90	90	90
γ/°	90	90	90	90	90
V/Å^3	360.70(19)	349.5(3)	341.4(3)	332.1(2)	327.2(2)
Z	4	4	4	4	4
Z'	0.5	0.5	0.5	0.5	0.5
Wavelength/Å	0.41	0.41	0.41	0.41	0.41
Radiation type	Synchrotron	Synchrotron	Synchrotron	Synchrotron	Synchrotron
Θmin/°	2.1183	2.1442	4.324	2.358	2.372
Θmax/°	20.4958	18.5811	18.227	20.515	20.674
Measured Refl.	816	362	339	642	631
Indep. Refl.	231	168	153	205	202
Refl. with I >2(I)	204	132	110	178	172
R_{int}	0.0156	0.0193	0.0153	0.0153	0.0456
Parameters	42	42	37	42	42
Restraints	0	0	0	0	0
Largest Peak	0.708	0.683	0.669	0.651	0.614
Deepest Hole	-0.511	-0.505	-0.51	-0.509	-0.457
GooF	0.857	3.0692	2.429	0.933	0.896
w_{R2} (all data)	0.1052	0.1365	0.1291	0.102	0.1088
w_{R2}	0.0985	0.1344	0.1251	0.101	0.1078
R_1 (all data)	0.0337	0.06	0.0662	0.034	0.0395
R_1	0.0318	0.0538	0.056	0.0335	0.0391

Table S2. Single crystal XDR refinement data of columbite-II CoV_2O_6 at selected pressures.

**Pressure (GPa)**	**20.0(1)**	**25.5(1)**	**31.3(1)**	**36.8(1)**	**43.5(1)**
Formula	CoV_2O_6	CoV_2O_6	CoV_2O_6	CoV_2O_6	CoV_2O_6
Dcalc./g·cm^{-3}	5.471	5.533	5.711	5.862	6.099
μ/mm^{-1}	2.367	2.388	2.466	2.54	2.638
Formula Weight	256.81	256.81	256.81	256.81	256.81
Colour	black	black	black	black	black
Shape	plate	plate	plate	plate	plate
Size/μm^3	20×20×5	20×20×5	20×20×5	20×20×5	20×20×5
T/K	293(2)	293(2)	293(2)	293(2)	293(2)
Crystal System	orthorhombic	orthorhombic	orthorhombic	orthorhombic	orthorhombic
Space Group	*Pbcn*	*Pbcn*	*Pbcn*	*Pbcn*	*Pbcn*
a/Å	12.617(6)	12.503(7)	12.403(7)	12.325(7)	12.278(7)
b/Å	5.2755(2)	5.2488(2)	5.2277(2)	5.2081(2)	5.1824(2)
c/Å	4.68420(10)	4.6611(2)	4.6391(2)	4.6200(2)	4.5984(2)
α/°	90	90	90	90	90
β/°	90	90	90	90	90
γ/°	90	90	90	90	90
V/Å^3	311.79(14)	305.89(17)	300.80(17)	296.56(17)	292.59(17)
Z	4	4	4	4	4
Z'	0.5	0.5	0.5	0.5	0.5
Wavelength/Å	0.41	0.41	0.41	0.41	0.41
Radiation type	Synchrotron	Synchrotron	Synchrotron	Synchrotron	Synchrotron
Θmin/°	2.414	2.43	2.44	2.45	2.46
Θmax/°	20.656	20.81	20.88	20.89	20.99
Measured Refl.	625	622	605	585	578
Indep. Refl.	204	197	187	176	179
Refl. with I >2(I)	192	184	177	165	164
R_{int}	0.0134	0.0115	0.0234	0.0297	0.0122
Parameters	42	42	42	37	37
Restraints	0	0	0	0	0
Largest Peak	0.651	0.576	0.603	0.555	0.576
Deepest Hole	-0.543	-0.527	-0.492	-0.514	-0.433
GooF	0.869	2.3391	2.0541	1.6883	1.8062
wR_2 (all data)	0.0899	0.0902	0.1062	0.1149	0.099
wR_2	0.0885	0.0897	0.1057	0.1139	0.0983
R_1 (all data)	0.0295	0.0386	0.0419	0.0442	0.0348
R_1	0.029	0.038	0.0412	0.0432	0.0334

Table S3. Single crystal XDR refinement data of the monoclinic δ-CoV_2O_6 at selected pressures.

**Pressure (GPa)**	**47.5(1)**	**49.0(1)**	**50.0(1)**
Formula	CoV_2O_6	CoV_2O_6	CoV_2O_6
Dcalc./g·cm^{-3}	6.265	6.273	6.299
μ/mm^{-1}	2.711	2.714	2.725
Formula Weight	256.81	256.81	256.81
Colour	black	black	black
Shape	plate	plate	plate
Size/μm^3	20×20×5	20×20×5	20×20×5
T/K	293(2)	293(2)	293(2)
Crystal System	monoclinic	monoclinic	monoclinic
Space Group	$P2_1/c$	$P2_1/c$	$P2_1/c$
a/Å	6.71(2)	6.734(19)	6.746(18)
b/Å	4.4428(9)	4.4257(5)	4.4055(4)
c/Å	4.5837(10)	4.5798(7)	4.5750(7)
α/°	90	90	90
β/°	95.22(8)	95.00(6)	95.22(5)
γ/°	90	90	90
V/Å^3	136.1(5)	136.0(4)	135.4(4)
Z	2	2	2
Z'	0.5	0.5	0.5
Wavelength/Å	0.41	0.41	0.41
Radiation type	Synchrotron	Synchrotron	Synchrotron
Θmin/°	3.692	3.7	3.711
Θmax/°	19.425	19.407	21.098
Measured Refl.	210	297	283
Indep. Refl.	108	106	111
Refl. with I >2(I)	96	90	104
R_{int}	0.0147	0.021	0.018
Parameters	38	38	38
Restraints	0	0	0
Largest Peak	0.58	0.479	0.4
Deepest Hole	-0.522	-0.543	-0.365
GooF	1.055	1.16	1.023
w_{R2} (all data)	0.1144	0.1249	0.103
w_{R2}	0.1136	0.123	0.0989
R_1 (all data)	0.0467	0.0466	0.0377
R_1	0.0441	0.042	0.0341

Table S4. Atomic positions determined at selected pressures for columbite CoV_2O_6.

Pressure (GPa)		0.0(1)	5.0(1)	9.5(1)	15.9(1)	19.0(1)
Co	x	0	0	0	0	0
	y	0.17485(6)	0.1730(2)	0.16901(8)	0.16887(9)	0.16790(11)
	z	0.25	0.25	0.25	0.25	0.25
V	x	0.16293(6)	0.1634(2)	0.16251(9)	0.16230(8)	0.16256(8)
	y	0.31897(6)	0.3201(2)	0.32158(8)	0.32154(9)	0.32192(11)
	z	0.74596(5)	0.74655(18)	0.74624(6)	0.74623(5)	0.74615(5)
O1	x	0.0969(3)	0.0977(13)	0.0935(5)	0.0938(6)	0.0931(7)
	y	0.3957(2)	0.3959(7)	0.3959(3)	0.3962(3)	0.3961(3)
	z	0.4423(2)	0.4379(10)	0.4361(3)	0.4361(3)	0.4353(2)
O2	x	0.0841(4)	0.0806(14)	0.0832(5)	0.0833(7)	0.0839(7)
	y	0.12505(16)	0.1236(7)	0.1192(2)	0.1189(2)	0.1178(2)
	z	0.8949(3)	0.8967(10)	0.9010(3)	0.9008(3)	0.9017(3)
O3	x	0.2541(4)	0.2569(13)	0.2556(5)	0.2559(7)	0.2548(7)
	y	0.13181(18)	0.1301(7)	0.1248(3)	0.1249(2)	0.1235(2)
	z	0.5938(2)	0.5919(10)	0.5886(4)	0.5886(2)	0.5874(2)

Table S5. Atomic positions determined at selected pressures for columbite-II CoV_2O_6.

Pressure (GPa)		20.0(1)	25.5(1)	31.3(1)	36.8(1)	43.5(1)
Co	x	0	0	0	0	0
	y	0.14096(12)	0.14054(12)	0.14001(14)	0.13982(15)	0.13939(12)
	z	0.25	0.25	0.25	0.25	0.25
V	x	0.16074(8)	0.16054(10)	0.16065(13)	0.16078(14)	0.16057(13)
	y	0.33467(13)	0.33601(13)	0.33704(16)	0.33779(17)	0.33845(13)
	z	0.74050(9)	0.74054(9)	0.74036(11)	0.74041(12)	0.74029(10)
O1	x	0.0854(10)	0.0854(11)	0.0825(15)	0.0829(9)	0.0829(7)
	y	0.3832(2)	0.3837(3)	0.3843(3)	0.3846(3)	0.3853(3)
	z	0.4215(3)	0.4202(5)	0.4191(5)	0.4175(5)	0.4175(5)
O2	x	0.0802(11)	0.0797(11)	0.0821(15)	0.0812(9)	0.0809(7)
	y	0.1180(2)	0.1176(3)	0.1166(3)	0.1160(3)	0.1156(3)
	z	0.9193(3)	0.9194(4)	0.9202(4)	0.9201(5)	0.9210(5)
O3	x	0.2536(11)	0.2549(12)	0.2517(15)	0.2525(8)	0.2531(7)
	y	0.1200(2)	0.1193(3)	0.1182(3)	0.1171(3)	0.1169(3)
	z	0.5840(3)	0.5831(5)	0.5823(5)	0.5812(5)	0.5805(5)

Table S6. Atomic positions determined at selected pressures for the monoclinic δ-CoV_2O_6.

Pressure (GPa)		47.5(1)	49.0(1)	50.0(1)
Co	x	0.5	0.5	0.5
	y	0.5	0.5	0.5
	z	0.5	0.5	0.5
V	x	0.1959(11)	0.1940(12)	0.1947(10)
	y	0.4604(4)	0.45985(4)	0.4599(3)
	z	0.8714(5)	0.8748(5)	0.8744(4)
O1	x	0.386(5)	0.373(6)	0.370(5)
	y	0.1865(18)	0.185(2)	0.1876(15)
	z	0.6755(19)	0.6697(16)	0.6726(13)
O2	x	0.245(5)	0.257(5)	0.259(4)
	y	0.6739(16)	0.6730(17)	0.6720(12)
	z	0.5660(19)	0.5616(14)	0.5665(12)
O3	x	-0.059(5)	-0.050(6)	-0.056(5)
	y	0.6348(15)	0.6350(17)	0.6350(14)
	z	0.7959(16)	0.7994(19)	0.7960(15)

Table S7. Infrared modes, wavenumbers and pressure coefficients corresponding to the DFT calculated active Infrared modes at ambient conditions for columbite CoV_2O_6. ω_0 is expressed in cm^{-1} (uncertainty of ±5%) and P in GPa.

Mode	ω_0	$\partial\omega/\partial P$	Mode	ω_0	$\partial\omega/\partial P$	Mode	ω_0	$\partial\omega/\partial P$
B_{3u}^{1}	Acoustic		B_{2u}^{4}	259	4.2(1)	B_{1u}^{9}	415	3.6(3)
B_{2u}^{1}	Acoustic		B_{2u}^{5}	273	5.0(1)	B_{2u}^{9}	452	2.6(1)
B_{1u}^{1}	Acoustic		B_{1u}^{5}	277	1.4(1)	B_{3u}^{10}	462	3.9(1)
B_{3u}^{2}	119	1.0(3)	B_{2u}^{6}	290	1.7(2)	B_{3u}^{11}	478	5.5(1)
B_{2u}^{2}	139	2.7(1)	B_{1u}^{6}	293	1.5(3)	B_{1u}^{11}	530	2.2(1)
B_{1u}^{2}	174	2.4(2)	B_{3u}^{6}	301	1.3(1)	B_{3u}^{12}	585	1.2(3)
B_{1u}^{3}	178	1.0(1)	B_{3u}^{7}	306	1.3(6)	B_{2u}^{11}	610	4.7(1)
B_{3u}^{3}	198	5.5(1)	B_{1u}^{7}	325	0.7(1)	B_{1u}^{12}	627	2.4(1)
B_{2u}^{3}	209	2.8(1)	B_{3u}^{8}	355	5.2(1)	B_{3u}^{13}	678	3.3(1)
B_{3u}^{4}	221	4.2(1)	B_{1u}^{8}	364	3.5(1)	B_{2u}^{12}	705	1.8(1)
B_{1u}^{4}	226	3.9(1)	B_{2u}^{7}	390	1.5(1)	B_{2u}^{13}	765	3.0(1)
B_{1u}^{5}	242	6.2(1)	B_{2u}^{8}	408	4.0(1)	B_{3u}^{14}	796	2.6(1)
B_{3u}^{5}	251	5.2(3)	B_{3u}^{9}	405	1.9(1)	B_{1u}^{13}	817	1.6(1)

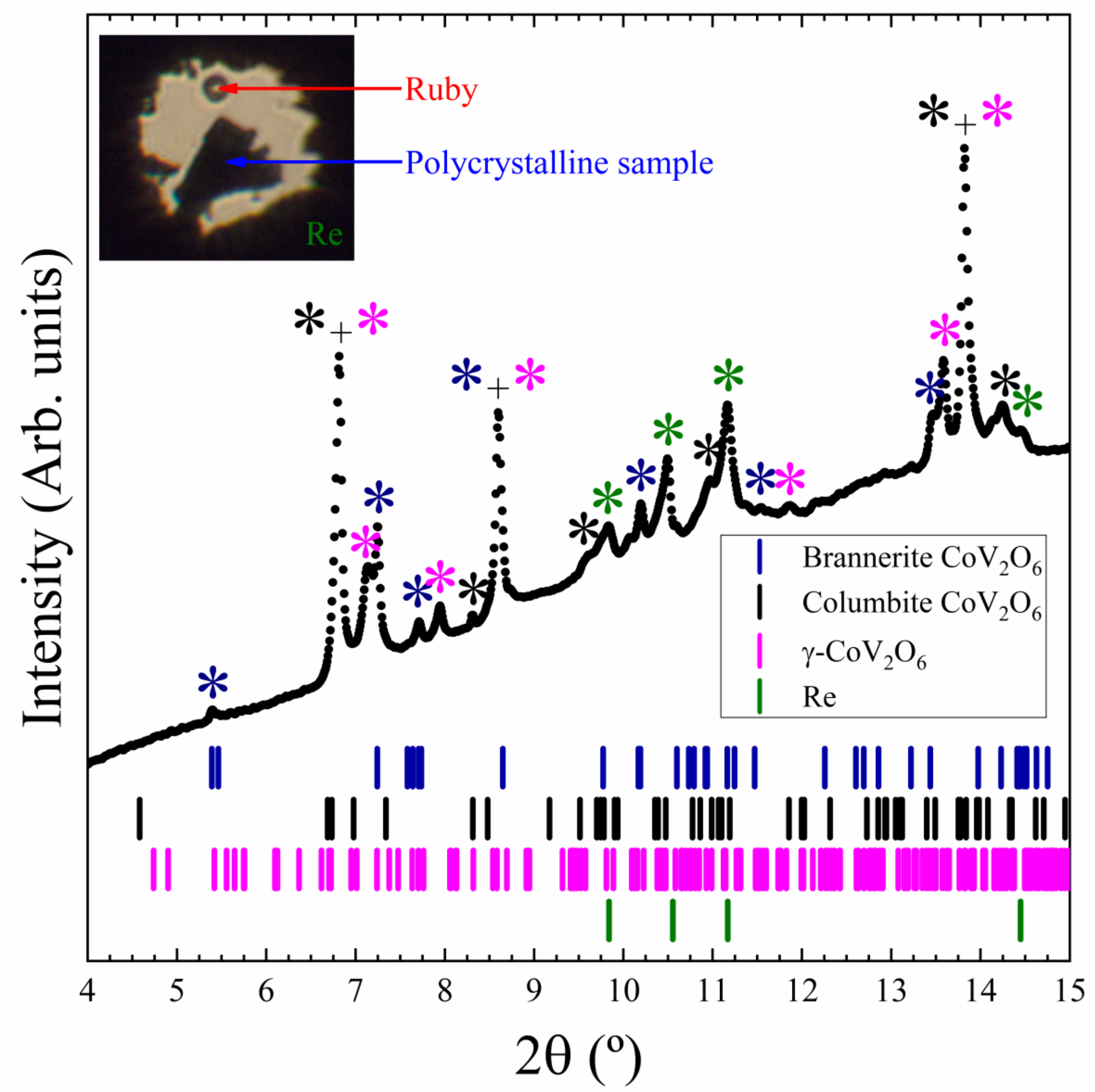

Figure S1. XRD measurement of the recovered sample after the Raman experiment on CoV_2O_6. The polycrystalline state of the sample and the mixture of phases make it impossible to carry out a refinement process, which renders this assignment tentative. The inset photo shows the state of the loading after the Raman experiment.
